# Validation of the accident and emergency experience questionnaire: a cross-sectional survey

**DOI:** 10.1186/s12913-023-09560-y

**Published:** 2023-06-09

**Authors:** Eliza Lai-Yi Wong, Annie Wai-Ling Cheung, Hong Qiu, Jonathan Chun-Hei Ma, Eng-Kiong Yeoh

**Affiliations:** Centre for Health Systems and Policy Research, JC School of Public Health and Primary Care, Faculty of Medicine, The Chinese University of Hong Kong, Hong Kong SAR, China

**Keywords:** Accident and emergency department, Accident and Emergency Experience Questionnaire, Exploratory factor analysis, Patient experience, Validation

## Abstract

**Background:**

Patient feedback is an important way for healthcare providers to understand patient experience and improve the quality of care effectively and facilitate patient-centered care in the healthcare system. This study aimed to suggest a validated instrument by evaluating the psychometric properties of the Accident and Emergency Experience Questionnaire (AEEQ) for measuring patient experience in the accident and emergency department (AED) service among the adult Chinese population.

**Methods:**

Attendances aged 18 or above from all public hospitals with AEDs during 16–30 June 2016 were targeted and a cross-sectional telephone survey was conducted using AEEQ. Preliminary AEEQ consisted of 92 items, including 53 core evaluative items and 19 informative items, and the other 20 items covered socio-demographics, self-perceived health status, and free open-ended comments on AED service. Psychometric properties of the evaluative items were evaluated for practicability, content and structure validity, internal consistency, and test-retest reliability in this study.

**Results:**

A total of 512 patients were recruited with a response rate of 54% and a mean age of 53.2 years old. The exploratory factor analysis suggested removing 7 items due to weak factor loadings and high cross-loading and then leaving 46 items grouped into 5 dimensions, which were care and treatment (14 items), environment and facilities (16 items), information on medication and danger signals (5 items), clinical investigation (3 items), and overall impression (8 items) to represent patient experience on AED service. The internal consistency and test-retest reliability were high with Cronbach’s alpha coefficient and Spearman’s correlation coefficient of the suggested scale of 0.845 and 0.838, respectively.

**Conclusion:**

The AEEQ is a valid and reliable instrument to evaluate the AED service which helps to build the engagement platform for promoting patient-centered care between patients and frontline healthcare professionals and improving healthcare quality in the future.

## Background

Patient feedback is an important way to understand what service users think about their care and treatment experience from the healthcare providers. Understanding patient experience represents an opportunity to elicit patients’ expectations and their perceived treatment’s effect, which could act as an indicator for evaluating and improving the quality of care [1]. The dynamic interactions between healthcare providers and patients in the healthcare system shape their attitudes so as to improve patients’ health outcomes [2]. Studies have shown that treatment adherence and clinical outcomes would be improved, and medical costs would be reduced due to positive patient experience [3]. On the contrary, a negative patient experience for individuals suffering from mental illnesses required further hospital readmission, subsequently leading to poor engagement and efficacy of care [4, 5].

Although there are different healthcare systems in countries worldwide, the accident and emergency department (AED) is an important place in the hospital to triage patients to have appropriate care and treatments afterwards, including in Hong Kong (HK) [6]. From most of the patients’ points of view, the visit to the AED also indicates their initial stage of the connections with the healthcare professionals in the healthcare system and as a starting point of their patient journey for the care in hospital. In order to provide effective care in AED, the collection of feedback or perception of performance from the patient perspective is essential information for quality of care improvement [7] which is a similar practice to other departments in hospitals [8–11]. Previous studies showed that patients admitted into the AED were usually distressed and confused, and issues that have long plagued emergency services, such as overcrowding, long waiting times, and poor communication continue to be the focus of patient experience research [12, 13]. These imply the importance of the collection of routine patient experience data. The collected information could be treated as a kind of patient measure to improve the quality of care effectively and facilitate patient-centered care in the healthcare system [14, 15].

Prioritizing quality improvement activities in the AED requires a validated and reliable instrument to collect the information from the patient [16]. Patient experience instruments specific to emergency services have been developed as early as 2003 in the UK, with institutions such as the Care Quality Commission (CQC) and the Picker Institute putting forth substantial effort in establishing a routine method for surveying feedback from AED patients [17, 18]. The US had also followed suit and established the Consumer Assessment of Healthcare Providers and Systems (CAHPS) survey to assess patient experience after the attendance of the AED since 2012 [19] and also other countries [20, 21]. A recent meta-synthesis indicated a framework for understanding the deteminants of patient experience in the AED [22]. In HK, the idea of understanding what patients think at a corporate-wide level has been started since 2009 [23]. Accidents and emergencies, inpatient and outpatient, usually contribute to major service delivery proportions in the healthcare system. Related patient experience measuring instruments for inpatient [9, 11] and specialist outpatient service [10] were developed in the local context respectively. Both instruments reported good validity and reliability for routine patient experience collection among HK Chinese population [9–11]. The instrument for AED service the ‘Accident & Emergency Experience Questionnaire (AEEQ) was identified as a strategic area for service improvement and followed to develop for assessing patient experience in AED service users in public hospitals [24] in HK. Thus, this study aimed at evaluating the psychometric properties of AEEQ for measuring AED service among the adult Chinese population. The findings could provide scientific evidence to determine the accuracy of measuring the target issues and confirm the completeness and consistency of the collected data [25]. It would establish a reference for its future refinement or revision and also a new development in other countries. Also, measuring patient experience is a valued step to promote patient-centred care in healthcare delivery.

## Materials and methods

### Study design and target population

This was a cross-sectional validation telephone survey using a structured questionnaire. Recruiting criteria were (1) HK residents; (2) aged 18 or above on the date of AED attendance during the sampling month; (3) able to be contacted within two weeks after attendance; and (4) Cantonese speaking. Patients who were known to be admitted to hospitals during the interview period were excluded from the survey. Proportional stratified hospital sampling was applied based on the overall attendance of all 17 public hospitals with AED to ensure the representativeness of the collected samples. The previous study has shown that a sample size of 300 is good and 500 is very good in a validation survey [26]. Therefore, we targeted a minimum of 500 eligible participants from the AED attendance which would provide adequate statistical power for the current study. Regarding the test-retest reliability, 50 respondents (around 10% of the overall respondents) from the validation survey were randomly selected and invited to complete the same questionnaire after their first interview two weeks later.

### Questionnaire development

Due to the comprehensiveness of the developed AED instrument for measuring patient experience applied in the UK national survey by Picker Institue Europe [27], it acted as the preliminary conceptual framework to derive the initial items of the questionnaire which could be included to local AED version for the Chinese population to indicate the quality of care. For the cultural adaptation of the instrument in HK, a total of 3 focus group discussions with 23 respondents who used the AED service during the survey period. The discussion findings provided additional local views and concerns based on important areas of the care ascpets in the quality of care they had received for the instrument development. Based on the findings, the preliminary framework of the questionnaire was constructed and further discussion with the target service provider and our international experts included the developer of UK national survey and the expert in the Pick Institute Europe who had rich experiences developed and conducted patient experience survey was made in confirming an initial AED patient experience questionnaire in the local context. Afterward, 10 individual cognitive debriefing interviews were performed to test the face validity of the questionnaire. It was used to evaluate the target users’ understanding of the proposed questionnaire and also confirm its feasibility and applicability of the developed AED instrument before proceeding to the psychometric validation. The participants in cognitive debriefing interviews found the questionnaire was clear, understandable, and appropriate. None of the participants commented that they found any of the questions offensive and uncomfortable. They also expressed that the length of the questionnaire was acceptable.

A preliminary questionnaire consisting of 92 items, 72 items capturing AED experience included 53 core evaluative items and 19 informative items constituting 9 sections: (1) arrival at the AED, (2) waiting at the AED, (3) hospital environment and facilities, (4) hospital staff, (5) care and treatment, (6) tests, (7) pain, (8) leaving AED, and (9) overall AED experience. Responses to the 53 evaluative items are used to indicate the direction for quality improvement of hospital service. The remaining 20 items covered socio-demographics, self-perceived health status, and free open-ended comments on AED service [22]. In addition, three demographic characteristics include age, gender and whether living in an old age home were retrieved from the hospital records for the comparison between respondents and overall attendance during the study period.

### Statistical analysis

Psychometric properties of 53 evaluative items were evaluated for practicability, content and structure validity, internal consistency, and test-retest reliability.

*Practicability of the questionnaire.*

The practicability was evaluated through the completion time to answer all the questions in the interview and the missing rate of each question [10, 11, 28]. The average completion time of all participants was used to check whether the length of the survey instrument was acceptable. For the questions that did not need to be answered or skipped by some patients, we recorded them as a new category as “0”. The questions that were refused to be answered or answered as ‘Don’t know/Forgot’, we looked at them as missing data. The missing rate of each question was calculated.

**Fig. 1:**
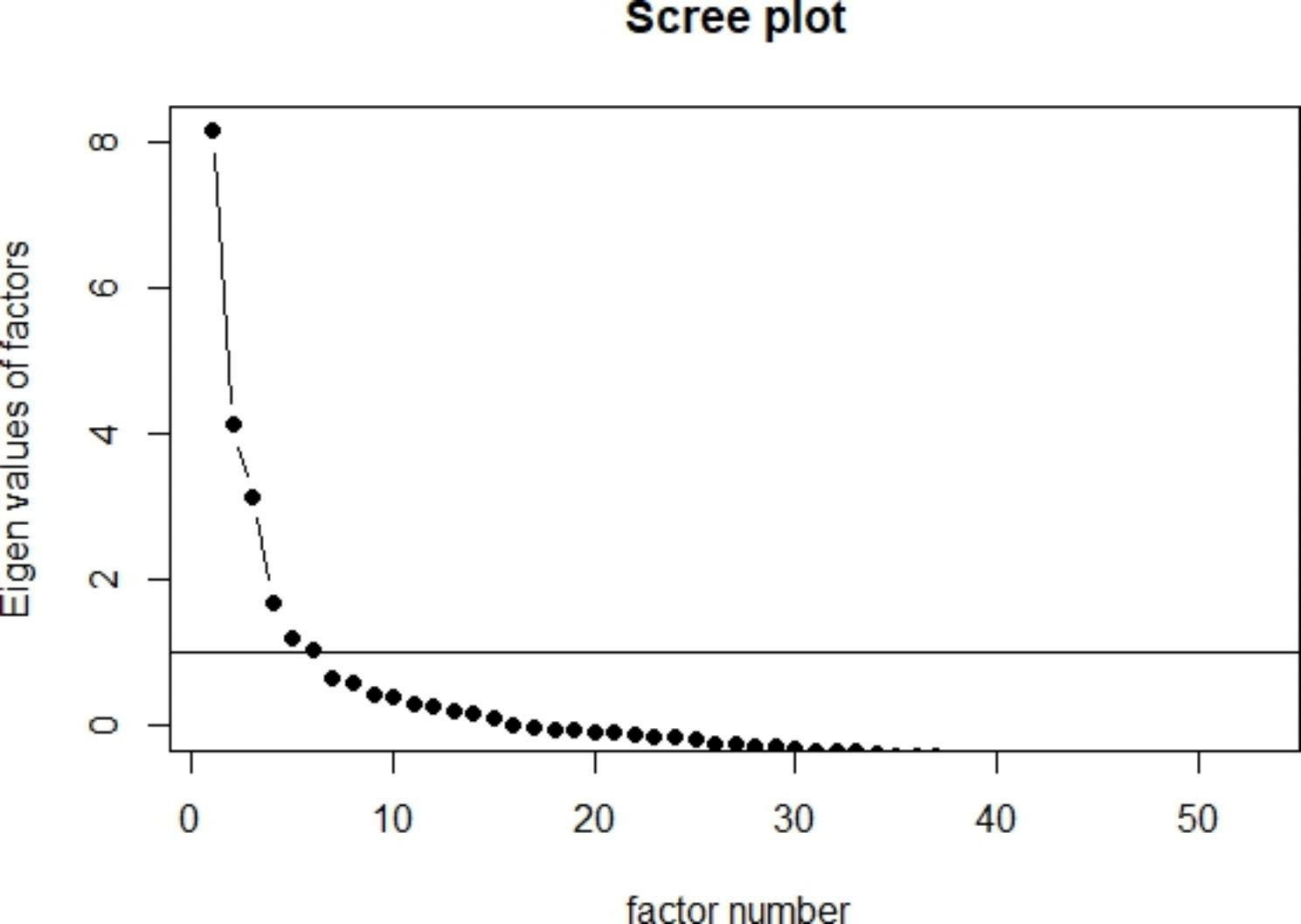
Scree plot to identify the number of factors

*Content validity.* We used the exploratory factor analysis (EFA) to test the internal structure of the AEEQ [29, 30]. Mardia’s multivariate normality test showed the data were not normally distributed, therefore we used the classical principal axis factoring which does not require normally distributed data [31]. Then we checked the suitability of data for an EFA through Kaiser-Meyer-Olkin (KMO) and Bartlet’s test [29]. In general, KMO measure great than 0.7 indicates the adequacy of the sampling, and a significant Bartlet’s test indicates the correlations between the items. The optimal number of factors was determined by Kaiser’s eigenvalue greater than 1 rule and Cattell’s scree test [32, 33]. The eigenvalue is interpreted as the proportion of the information in a factor. The cut-off of 1 means the factor contains information equal to 1 item, thus it is not worthwhile keeping a factor with information less than 1 item. Based on the eigenvalues of factors and the scree plot (Fig. 1), we judged the suitable number of factors for this AEEQ is five or six.

As our data are not normally distributed, we used the classical principal axis factoring to extract the pre-defined five factors [31, 34]. Following our previous study, we used Promax oblique rotation to obtain clear factors and factor loadings in the EFA [10], which “anticipated interrelationships between the latent factors in the model and to generate a more realistic approximation of the true relationships between items” [34]. Factor loadings (FLs) show the item-factor relationship with partial correlation coefficients of factors to items and a value of less than 0.3 was looked as poor loading. Item complexity is another indicator to judge if the item is specific to a factor (should have an item complexity close to one). Item complexity of much greater than one means cross-loading. An item with poor FLs and cross-loading was suggested to be removed from the questionnaire [31]. We removed one item at a time with the poorest FLs and the highest complexity and repeated the factor analysis until all the remained items had acceptable FLs and were specific to a factor. The correlation matrix among the proposed factors and the proportion of the variance explained by each factor was examined [29, 33].

*Reliability.* Internal reliability (internal consistency) of the factors extracted from the EFA was evaluated by Cronbach’s alpha coefficient. We determined the reliability of each factor separately by including the selected items per factor. Cronbach’s alpha coefficient, ranging from 0 to 1, serves as the indicator of the internal reliability of an instrument. A coefficient of great than 0.7 indicates respectable internal consistency. We also evaluated the external reliability of the instrument by using the questionnaire two weeks later after the first survey with a randomly selected 10% of the sample population [10, 11]. In the current study, 50 patients were randomly selected for the test-retest reliability survey and invited to complete the same questionnaire after their first interview. The test-retest consistency was assessed by Spearman’s rank correlation coefficient and corresponding significance test for each dimension of the questionnaire.

All analyses were performed using R statistical environment version 4.1.2. with a ‘psych’ package for exploratory factor analysis [35, 36]. All tests were two-sided and P < 0.05 was considered statistically significant.

### Ethical considerations

Ethical approval was obtained from The Joint Chinese University of Hong Kong – New Territories East Cluster Clinical Research Ethics Committee. Verbal consent was obtained by the trained research staff before the commencement of the telephone interview. All respondents were informed about the purpose of the study, research procedures, and their rights within the study before the interview were conducted. Participants were allowed to refuse to answer questions or withdraw from the study at any time point. All the information was anonymous and was treated with strict confidentiality.

## Results

### Characteristics of study subjects

A total of 512 patients were recruited for the validation survey and completed the interview with a response rate of 54%. The mean age of the 512 participants was 53.2 years old (SD = 18.7), among which 46.5% were men. Compared with the corresponding 43,904 AED attendances during the same study period, the respondents were younger (p < 0.05), comparative gender proportion (p = 0.49), and fewer lived at the old age homes (p < 0.05) (Table 1). Additionally, around half of the participants had an education of secondary level, were full-time or part-time workers, and 26.6% of them were receiving government allowance such as comprehensive social security assistance, disability allowance, or old age allowance. 68% of them attended the AED only once in the past 12 months, and in general, 52.5% of them thought they were in good health status (Table 1).

**Table 1:** Demographic characteristics ^a^ between participants and corresponding AED attendance during the study period

Demographic characteristics	Participants		AED attendance		P-value ^b^
	N = 512	%	N = 43,904	%	
Age, Mean ± SD	53.2 ± 18.7	-	55.2 ± 20.3	-	0.028*
Gender, Men	238	46.5	21,081	48.0	0.485
Living in old age home	3	0.6	1862	4.2	< 0.001*
Education level			NA		NA
Primary and below	162	31.6			
Secondary	249	48.6			
Tertiary and above	99	19.3			
Working status			NA		NA
Retired	162	31.6			
Unemployed	18	3.5			
Full-time student	11	2.1			
Housewife	64	12.5			
Full-time worker/ Part-time worker	254	49.6			
Receiving government allowance ^c^	136	26.6	NA		NA
AED attendance in the past 12 months			NA		NA
Only 1 time	348	68.0			
2–3 times	144	28.1			
4–5 times	13	2.5			
>=6 times	7	1.4			
Self-report general health status			NA		NA
Very good	14	2.7			
Good	269	52.5			
Fair	182	35.5			
Poor	44	8.6			
Very poor	3	0.6			

^a^: Only three items of demographic characteristics (age, gender, and whether living in an old age home) were retrieved from the hospital records for the comparison between respondents and overall attendance during the study period; others were provided by the participants through the AEEQ survey only

^b^: T-test was carried out to continuous variables such as age, and chi-square tests were carried out to other categorical variables

^c^: Types of the government allowance included (i) Comprehensive Social Security Assistant, (ii) disability allowance, and (iii) old age allowance

*: P-value is statistically significant at 0.05 level

### The practicability of the questionnaire

The interview took approximately 18 (± 3) minutes on average to complete. More than half of the respondents (51.0%) spent 18 min or less and around 80.7% spent 20 min or less. There were no incomplete interviews. The missing rate of each question ranged from 0 to 2.15%.

### Validity

Fifty-three items in the questionnaire were evaluative and included in the exploratory factor analysis. The value of the KMO test was 0.830, which indicated some underlying common factors in the matrix. Bartlett’s test of sphericity (P < 0.05) showed that the matrix was significantly correlated. Kaiser’s eigenvalue greater than 1 rule and Cattell’s scree test identified that the number of factors would be five or six (Fig. 1). Then we performed EFA with fixed five factors. Some items with weak factor loadings (FLs < 0.3) and a high cross-loading index (Complexity > 2) were systematically removed one by one in each repeated EFA. The item *“was it easy to get through the main entrance and move around in the A&E Department”* was removed firstly from the analysis because of FLs < 0.3 and high complexity of 3.64. Six more items were removed step by step based on the same criteria. The remaining 46 items were grouped into 5 dimensions, with all FLs > 0.3 and a mean item complexity of 1.3. Each factor explained the proportion of variance by 24.3%, 25.2%, 22.5%, 17.8%, and 10.3%, respectively, showing the relative importance of each extracted dimension. A 6-factor model was also reviewed, while the 5-factor model had the least cross-loading and was the most conceptually interpretable given by the experts.

The included questions in the five extracted dimensions with FLs and complexity are shown in Table 2. The extracted dimensions were named “overall impression” (8 items), “care and treatment” (14 items), “environment and facilities” (16 items), “information on medication and danger signals” (5 items), and “clinical investigation” (3 items), respectively, representing the content and internal structure of the AEEQ. The correlation coefficients among the five dimensions ranged from 0.036 to 0.510, showing that the factors were distinct from each other. Seven questions due to the lower level of FLs were suggested to remove and displayed in Table 3. It was approved by our experts as the information collected from five of the seven deleted items (such as “*was it easy to get through the main entrance and move around in the A&E Department”*, *“was the A&E Department the right temperature for you”, “overall, did you feel that you had to wait a long time for all your care processes in A&E”, “if you needed assistance, were you able to get a member of medical or nursing staff to help you”, “while in the A&E Department, did you ever see any posters or leaflets explaining how to complain about the care you received”*) has been captured from the remaining items, or there were a high proportion of participants who skipped the other two items (38% skipped “*did you feel that you had to wait a long time for a triage nurse to assess your priority*” and 58% skipped “*do you think the hospital staff did everything they could to help control your pain*”).

**Table 2:** Suggested dimensions with factors loadings (FL) and complexity (Com) of the items from the exploratory factor analysis* for the AEEQ

Suggested dimensions and included questions	FL	Com^#^
**Overall Impression (8 items)**		
27) Did you have enough time to discuss your health or medical problem with the doctor?	0.532	1.60
59) At any point, did you ever feel worried that staff in the A&E Department had forgotten about you?	-0.535	1.16
61) Was the main reason you went to the A&E Department dealt with to your satisfaction?	0.642	1.12
63) Overall, did you feel you were treated with respect and dignity while you were in the A&E Department?	0.509	1.21
64) Did you have confidence and trust in the doctors treating you?	0.817	1.07
65) How would you rate the care you received from the doctors?	0.819	1.01
66) Did you have confidence and trust in the nurses treating you?	0.778	1.18
67) How would you rate the care you received from the nurses?	0.773	1.03
**Care and Treatment (14 items)**		
11) Did you feel that you had to wait a long time before being seen by a doctor?	0.304	1.80
28) While you were in the A&E Department, did a doctor explain your condition and treatment in a way you could understand?	0.583	1.73
29) Did you know of the name of the doctor treating you?	0.631	1.70
30) If your family or someone else close to you wanted to talk to a doctor, did they have enough opportunity to do so?	0.623	1.27
31) Did doctors and other hospital staff talk to each other as if you weren’t there while discussing your condition with you?	0.642	1.88
32) Did the hospital staff listen to what you had to say?	0.477	1.64
33) If you had any worries or fears about your condition or treatment, did hospital staff discuss/ comfort you about your condition?	0.434	1.06
34) In your opinion, was the hospital staff aware of your condition or treatment?	0.585	1.80
35) While you were in the A&E Department, were you given enough information about your condition or treatment?	0.412	1.92
36) Was enough information about your condition or treatment given to your family or someone close to you?	0.562	1.75
37) If you had important questions to ask a doctor about your care and treatment, did the doctor provide a clear and understandable answer to you?	0.677	1.54
38) If you had important questions to ask a nurse about your care and treatment, did the nurse provide a clear and understandable answer to you?	0.611	1.38
39) Were you given enough privacy when discussing your condition or treatment/ being examined or treated?	0.445	1.02
40) Were you involved in decisions about your care and treatment?	0.584	1.09
**Environment and Facilities (16 items)**		
3) Once you arrived at the hospital, was it easy to find your way to the A&E Department?	0.407	1.14
4) How would you rate the courtesy of the staff at the A&E Department registration counter?	0.364	1.28
8) Did the hospital staff provide any information on how long you would have to wait?	0.492	1.04
9) Did the hospital staff give enough privacy when discussing your condition in the triage station?	0.321	1.39
13) In your opinion, how clean was the A&E Department (except toilet)?	0.300	1.32
14) How clean were the toilets in the A&E Department?	0.415	1.46
15) Were you able to find a place to sit in the A&E department?	0.502	1.24
16) How was the air ventilation/ circulation in the A&E Department?	0.341	1.34
18) Did you see any signs in A&E asking patients with fever to sit in the fever area?	0.486	1.24
20) Did you see any posters or leaflets in the A&E Department asking patients and visitors to wash their hands or to use hand-wash liquid/ gels?	0.625	1.06
21) Were hand-wash liquid/ gels available for patients and visitors to use?	0.643	1.09
22) Did you see any posters or leaflets in the A&E Department asking patients and visitors to wear a mask in the A&E Department?	0.647	1.17
23) Were surgical masks available (including free or paid) for patients and visitors to use in the A&E Department?	0.622	1.03
24) While you were in the A&E Department, did you feel bothered or threatened by other patients or visitors?	0.426	2.44
25) Were you able to get suitable food or drinks when you were in the A&E Department?	0.421	1.14
26) If you needed to go to other parts of the hospital, was it easy to find your way around?	0.528	1.20
**Information on Medication and Danger signals (Homecare information) (5 items)**		
54) Did a member of staff explain to you how to take the medications?	0.936	1.02
55) Did a member of staff explain the purpose of the medications you were to take at home in a way you could understand?	0.953	1.04
56) Did a member of staff tell you about medication side effects to watch for?	0.867	1.01
57) Did the hospital staff provide clear information about your medicines (included written or printed)?	0.624	1.13
58) Did a member of staff tell you about what danger signals you should watch for after you went home?	0.582	1.26
**Clinical Investigation (3 items)**		
43) Did a member of staff explain why you needed these test(s) in a way you could understand?	0.824	1.03
45) Did you feel that you had to wait a long time for your test(s) to be carried out?	0.604	1.20
46) Did a member of staff explain the results of the tests in a way you could understand on the day you visited the A&E Department?	0.777	1.05

*: Principal axis factoring with Promax oblique rotation was used in this exploratory factor analysis. ^#^: complexity, an item specific to a factor should have a complexity close to one. The mean item complexity for all dimensions is 1.3

**Table 3:** Items suggested to be removed from the exploratory factor analysis

Items removed*	FL	Com
12) Was it easy to get through the main entrance and move around in the A&E Department?	< 0.3	3.64
60) Overall, did you feel that you had to wait a long time for all your care processes in A&E?	< 0.3	2.92
48) Do you think the hospital staff did everything they could to help control your pain?	< 0.3	2.42
17) Was the A&E Department the right temperature for you?	< 0.3	2.23
62) If you needed assistance, were you able to get a member of medical or nursing staff to help you?	< 0.3	4.13
69) While in the A&E Department, did you ever see any posters or leaflets explaining how to complain about the care you received?	< 0.3	2.65
6) Did you feel that you had to wait a long time for a triage nurse to assess your priority?	< 0.3	3.61

*: Items were removed one by one due to the poorest factor loading (< 0.3) and the highest cross-loading index (Complexity > 2) at each repeated EFA until the remaining items had acceptable FLs and were specific to a factor

### Reliability

The Cronbach’s alpha coefficient (α) of the overall scale was 0.845 and that of the five dimensions ranged from 0.748 to 0.862 (Table 4). Spearman’s rank correlation coefficient (ρ) for the test-retest consistency for the overall scale was 0.838 and that of the five extracted dimensions ranged from 0.761 to 0.955 with statistical significance (P < 0.001) (Table 4).

**Table 4:** Internal and external reliability for all and individual proposed five dimensions

Dimensions	Items included in each dimension	Internal consistency (n = 512)	Test-retest reliability (n = 50)
		Cronbach’s alpha coefficient α (95% CI)	Spearman’s rank correlation coefficient (ρ)
All dimensions	46	0.845 (0.829, 0.867)	0.838*
Each dimension			
Overall Impression	8	0.771 (0.748, 0.794)	0.761*
Care and Treatment	14	0.827 (0.806, 0.849)	0.826*
Environment and Facilities	16	0.800 (0.776, 0.824)	0.774*
Information on Medication and Danger Signals	5	0.862 (0.844, 0.880)	0.995*
Clinical Investigation	3	0.748 (0.712, 0.784)	0.931*

*: p-value for the Spearman’s ρ is statistically significant at 0.001 level

## Discussion

A comprehensive instrument measuring patient experience on AED service (AEEQ), was validated among the adult Chinese population. The analysis reported the psychometric properties of the developed instrument and showed evidence that the practicability, validity, and reliability of AEEQ. Patients averagely spent a reasonable time (around 20 min) to complete the questionnaire by telephone interview with a low missing rate of each question. It indicates that the performance of AEEQ is practicable and concise which is similar to those locally validated instruments for measuring patient experience among adult HK Chinese population in different settings [9–11]. The high responses for each evaluative question also imply good acceptability of these tools and the questions are understandable to answer. It has similar or even better performance when compared with other validated instruments for patient experience surveys [7, 28]. The validated AEEQ also could act as a reference for other jurisdictions [16].

The factor analysis suggested 5 dimensions to represent patient experience on AED service using the AEEQ and the proposed dimensions included: “care and treatment”, “environment and facilities”, “information on medication and danger signals”, “clinical investigation”, and “overall impression”. The suggested dimensions are comparable to the framework based on the meta-synthesis [22] and other validated instruments for the patient-reported experience measures for AED service [7, 20, 28, 37]. The suggested multi-dimensional patient experience model also shows good internal consistency and external reliability. It suggests that the instrument has high construct validity for measuring care aspects of patient experience in accident and emergency service. Thus, the instrument could provide a direction to obtain a summary index to show the performance of AED service [7] for routine patient experience collection and comparison over time. The confirmed version of the AEEQ almost covered all care aspects suggested in the UK studies [7, 28]. Similarity, Our study also highlighted the importance of patient-healthcare staff interaction including the information given for treatment or discharge and the care and treatment received from the healthcare workers which are echoed by the overseas studies [7, 20]. Interestingly, exploratory factor analysis excluded the seven items related to waiting time, pain control, get help, feedback channel and get access with factor loading less than 0.3, these areas are covered in the UK studies [27]. The benefit of removing unnecessary items in order to have more concise instruments for the operation of data collection [20]. For the face content analysis, experts decided to retain seven items in the instrument in the first benchmark survey because these areas are recognized as one of the patient-centered aspects in oversea and it may enable us to improve and benchmark the quality of care.

There were some limitations in the study. The participants who were recruited for the validation survey were significantly younger and less likely to live in an old age home than the general AED attendance population. Although the response rate was similar when compared to other local patient experience surveys [10, 11] or overseas applying the postal survey [7, 28] it should be cautious that we may not reach the patients who were in poor health or who lived in an old age home during the study. Thus, further studies are needed to explore AED experience of elders who live in the old age home using other survey channels. Then, all of our recruited respondents were AED users in public hospitals. Although the instrument has covered all the core care aspects for public AED service, review and revision should be conducted before applying to those who attended AED in a private setting. But it still could be a good reference in general for those with similar care aspects in the patient journey.

## Conclusions

The present study provided evidence of the practicability, validity, and reliability of the patient experience questionnaire for routine patient experience collection and comparison over time for the accident and emergency service. The findings could serve as a recommendation for essential practices from improve patient experience. The instrument also makes it possible to build the engagement platform for promoting patient-centered care between patient and frontline health professionals and improving healthcare quality in the future.

## Data Availability

The data that support the findings of this study are available from Hong Kong Hospital Authority but restrictions apply to the availability of these data, which were used under license for the current study, and so are not publicly available. Data are however available from the correspondence author – Professor Eliza Wong upon reasonable request and with permission of Hong Kong Hospital Authority.
